# Directed forgetting of pictures of everyday objects

**DOI:** 10.1167/jov.22.10.8

**Published:** 2022-09-09

**Authors:** Paul S. Scotti, Ashleigh M. Maxcey

**Affiliations:** 1 Department of Psychology, The Ohio State University, Columbus, OH, USA; 2 Department of Psychology, Vanderbilt University, Nashville, TN, USA

**Keywords:** visual long-term memory, directed forgetting, voluntary control, long-term memory

## Abstract

Directed forgetting is a laboratory task in which subjects are explicitly cued to forget certain items and remember others. Volitional control over the contents of memory has been used to study clinical disorders, with successful intentional control of memory being a hallmark of a healthy mind. Yet the degree of volitional forgetting over the content of visual long-term memory is unclear when compared to words. Different kinds of visual stimuli (e.g., abstract symbols, line drawings, scenes) may not equally be susceptible to voluntary control in memory, and intentional forgetting studies have shown both twice as much forgetting of pictures compared to words (think/no-think task) and half as much forgetting of pictures compared to words (directed forgetting task). In the present study, we systematically test volitional control over pictures of everyday objects using item-method directed forgetting procedures. We find that subjects are able to intentionally prioritize memory for pictures cued as to be remembered over pictures cued to be forgotten. Here we show that directed forgetting effects are observed using pictures of everyday objects (albeit to a weaker extent compared to directed forgetting of words), suggesting increased confidence for generalization of directed forgetting literature to real-world applications. However, we caution clinical applications of intentional memory control until the underlying direction causing this effect (upregulation of remember-cued items or downregulation of forget-cued items) is known.

## Introduction

Humans tend to believe that they have a degree of volitional control over their ability to forget unwanted memories. For example, students ask professors if material will be on the exam to determine what they can forget, and judges direct juries to disregard information that was inappropriately introduced into a trial. The ability to volitionally forget has been characterized as a hallmark of well-being that is impaired in anxious and depressed individuals (Hauswald & Kissler, 2008; Stramaccia et al., 2021), and intentional forgetting tasks have often been used to study cognitive effects of clinical disorders, including obsessive-compulsive disorder and posttraumatic stress disorder (Delaney et al., 2020).

Intentional forgetting can be studied in the laboratory using a variety of methods (Maxcey & Woodman, 2014a; Scotti & Maxcey, 2021). In the *think/no-think* procedure (Anderson & Green, 2001), subjects learn word pairings such that one word in the pair cues recall of the other word. Then one word in the pair is presented and followed by a cue to either think of the other word in the pair (the recall condition) or not think of the other word (the suppress condition). In a final memory test, participants are typically worse at retrieving suppressed pairs relative to recalled pairs, suggesting successful suppression-induced forgetting (Anderson & Huddleston, 2012; Hertel & McDaniel, 2010). In the *directed forgetting* procedure, explicit cues direct subjects to forget some of the presented information while remembering the rest (Bjork, 1972; Bjork et al., 1968; Geiselman et al., 1983; MacLeod, 1998; Sheard & MacLeod, 2005; Thompson et al., 2011). In one such method, a subject is given a list of items (often ranging from 12–16 items per list; Sahakyan & Foster, 2016) to remember and is later told whether that specific list should be remembered or forgotten. Another method, the item method, presents items sequentially, with each item followed by the direction to remember or forget (MacLeod, 1998). The directed forgetting effect is shown when subjects perform as instructed, with better memory for remember-cued items compared to forget-cued items, suggesting subjects are exerting control over the contents of memory and forgetting the information as instructed (Anderson & Hanslmayr, 2014; Bjork, 1972; Chiu et al., 2021; Johnson, 1994; Sheard & MacLeod, 2005).

Intentional forgetting of pictures appears robust in the think/no-think procedure, where the size of the forgetting effect is doubled when using pictures compared to words (Stramaccia et al., 2021), leading to a small to moderate effect^1^ of suppression-induced forgetting of pictures and a small effect of suppression-induced forgetting of words. However, intentional forgetting of pictures appears weak in the directed forgetting procedure, where forgetting is reduced by more than 60% with pictures relative to words (Quinlan et al., 2010). This inverse relationship between the ability to intentionally forget information depending on stimulus type calls into question the extent to which humans can intentionally control forgetting. The instruction to suppress information in the think/no-think procedure involves an association between paired stimuli, suggesting directed forgetting is a purer measure of the ability to volitionally forget visual information. Further complicating matters, unintentional forgetting (Maxcey & Woodman, 2014b) causes deeper forgetting of pictures than intentional forgetting (Scotti & Maxcey, 2021), and in some circumstances, only the cue to remember, not the cue to forget, has an effect (Sunby et al., 2019; Tozios & Fukuda, 2020). These results show that, despite the proposed relationship between directed forgetting and mental health (Hauswald & Kissler, 2008; Stramaccia et al., 2021), the direction of directed forgetting over pictures in visual long-term memory that should be predicted in the typical subject population is unknown (Tozios & Fukuda, 2020).

### Present study

Here we determine the degree of directed forgetting over memory for pictures of everyday objects stored in visual long-term memory. The type of pictures applied in directed forgetting procedures to study volitional control over visual memory has varied from abstract symbols (Hourihan et al., 2009) to line drawings (Quinlan et al., 2010) to entire scenes (Hauswald & Kissler, 2008). Unlike existing studies, here we use pictures of everyday objects on a white background (Scotti & Maxcey, 2021) to balance control over the stimuli and ecological validity in understanding the nature of top-down control over visual memory. Further, everyday objects represent a good intermediate stimulus between low-level/abstract visual stimuli and high-level/real-world visual scenes (see Brady et al., 2019), allowing us to compare directed forgetting magnitudes across the scale of simple to complex settings.

Systematically studying memory across different memoranda is important for developing accurate models of memory. For example, according to signal-detection theory, recognition memory performance is impacted by a signal and noise (Green & Swets, 1966; Wickens, 2002). Although much research has focused on the nature of the signal, less is understood about the source of the noise. The source of noise appears to differ across memoranda, with background noise dominating memory for words (Osth & Simon, in press) and item noise driving memory for fractals and nonfamous faces (Osth & Dennis, 2015). Given that previous research has shown moderate activation to be a key determinant in eliciting forgetting (Detre et al., 2013; Lewis-Peacock & Norman, 2014; Norman et al., 2007), different memory stimuli evoking different sources of noise with varying signal-to-noise ratios may substantially impact whether voluntary control of memory contents is observed.

Across the first two experiments, we implemented one standard and one modified directed forgetting procedure by varying available responses including a tagging procedure (Corenblum et al., 2020; Goernert et al., 2011; Goernert et al., 2021; Goernert et al., 2007; MacLeod, 1999; Otani et al., 2012; Thompson et al., 2011). The tagging method offers subjects an extra response option to report that they remember an item but they also remember that it was cued to forget. Tagging can reduce response confusion and ensure a purer measure of directed forgetting, as it should prevent subjects from mistakenly believing that they should report an Old item as New because they remember that it was previously studied with the instruction to forget (these cases would spuriously inflate directed forgetting effects). The tagging procedure has been employed in recognition item-method directed forgetting tasks with words (Thompson et al., 2011) and faces (Corenblum et al., 2020) but not pictures of everyday objects.

**Figure 1.**
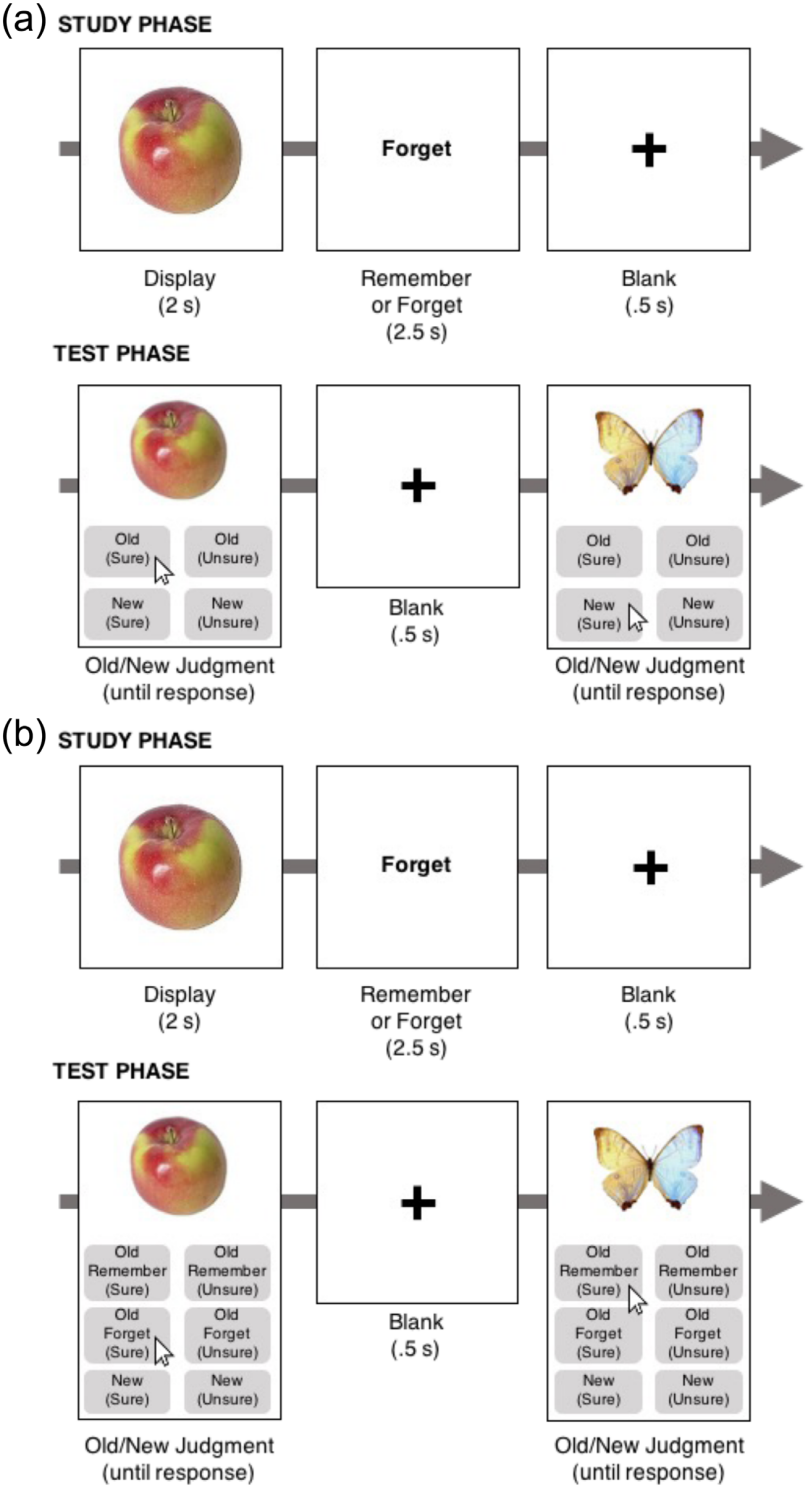
Methods in (a) Experiment 1 and (b) Experiment 2. The experiment began with a 100-item study phase. Half the objects were followed by the cue to Forget and half were followed by the cue to Remember. In the test phase, subjects had either (a) four possible response buttons to select from to report the item was Old or New, followed by Sure or Unsure, or (b) six possible responses because Old responses were further divided by whether the item was cued to Remember or cued to Forget, followed by Sure or Unsure.

Experiment 1 emulated the typical responses available in a directed forgetting experiment (Old or New, further subdivided by Sure and Unsure confidence ratings, resulting in four total response options; Figure 1a). Experiment 2 provided an additional response by subdividing the responses to Old items by Old-Cued-to-Forget and Old-Cued-to-Remember (further subdivided by Sure and Unsure confidence ratings, resulting in six total response options; Figure 1b). Postexperiment debriefing followed both experiments to assess the proportion of subjects who report believing the instruction to forget. Then we asked whether directed forgetting differed across subjects who did and did not believe in the instruction to forget. If voluntary control over forgetting of pictures of everyday objects is present and predictable (Corenblum et al., 2020; Quinlan et al., 2010; Thompson et al., 2011), then both of the experiments will result in reliable directed forgetting.

Our final experiment aims to further clarify the role of demand characteristics and directly compare directed forgetting effects between pictures of everyday objects and verbal words within the same experiment. An argument can be made that the online environment of Experiments 1 and 2 may introduce additional factors that are unaccounted for in initial analyses. To clarify the validity of the observed directed forgetting effects, we replicate the modified directed forgetting procedure from Experiment 2 in a laboratory setting using a within-subjects design to test directed forgetting of both pictures and words. If the directed forgetting effects in a controlled laboratory setting testing both pictures and words are comparable to the effects observed in the online experiments, then the validity of the results of Experiments 1 and 2 will be bolstered. Further, such a within-subject design allows us to directly compare the magnitudes of directed forgetting across stimulus types.

## Experiment 1: Traditional directed forgetting

### Methods

#### Participants

The first demonstration of item-method directed forgetting was conducted by Muther (1965) with words. Effect size was not reported, but Muther reported a *p* value of <0.001 with 12 participants. However, previous studies finding minimal directed forgetting of pictures (Quinlan et al., 2010) suggest a larger sample size is useful to detect evidence of directed forgetting of pictures. Data were collected online using Amazon Mechanical Turk (MTurk). Online subject recruitment necessitates quality assurance checks to ensure data were worthy of further analysis. Across both experiments, we excluded subjects who performed at or below chance (less than or equal to 50% accuracy and/or more than or equal to 50% false alarms). This resulted in different sample sizes across experiments. Rather than reduce each experiment to the smallest sample size for the sake of parsimony, we report all data that passed the initial quality assurance checks to most powerfully capture the potentially small effects of interest. All data are provided online. Subjects were not screened for color blindness or visual acuity impairments and were compensated at $6/hour. The Vanderbilt University Institutional Review Board approved all procedures.

Data were collected from 100 subjects. Quality assurance trimming left 62 subjects remaining (mean age: 29.65 [*SD* 3.87], 25 female, 36 male, 1 preferred not to answer) who were included in analyses below.

#### Quality control measures for online data collection

In addition to the exclusion criteria outlined above, the following measures were implemented to control the quality of online data collection. At the end of the experiment, subjects had to complete a simple captcha test before submitting the data. The test comprised typing a random sequence of letters into a textbox field. In addition, MTurk workers (i.e., participants) had to pass all of the following criteria to be eligible to participate: 18–35 years old, live in the United States, hold an MTurk approval rating of ≥ 98%, successfully have completed over 750 MTurk tasks prior to this experiment, and cannot have participated in a previous MTurk experiment run by our lab. We did not award any bonus based on performance, so there was no incentive for using aids like taking cell phone pictures of the items, which would be more effort than it is worth. Finally, in Experiment 3, we replicated the results of Experiment 2 in a controlled laboratory setting, confirming the results collected online in Experiments 1 and 2.

#### Stimuli

Experiments were conducted online using MTurk, meaning that monitors could vary in size, viewing distance, color calibration, and so on. We report stimulus sizes in pixels and not degrees of visual angle because of these variable environments. All stimuli were displayed inside of a 600-px × 600-px white square, with 300-px × 300-px items presented in the center of the square. The total stimulus set consisted of 200 pictures of everyday objects presented on a white background. Stimuli, originally from (Brady et al., 2008), are available online (http://github.com/maxceylab/maxceylab.github.io/tree/master/stimuli/unique). All objects were unique and did not share a common object category. The assignment of pictures to remember cue, forget cue, or lure was random for each subject.

#### Procedure

The procedure for Experiment 1 is shown in Figure 1a. The study phase included 100 total trials (50 cued to remember, 50 cued to forget) composed of unique real-world objects. Subjects were instructed to study the visual details of each object. Each object was displayed for 2 seconds, followed by the word *remember* or *forget* displayed for 2.5 seconds, directing them to either forget an object or remember the object for a later memory test.

To better ensure that we are testing long-term memory representations, participants underwent a 2-minute filler task between the study and test phases. The filler task was a simple color change detection task using colored squares (e.g., Luck & Vogel, 1997) where participants were briefly shown an array of colored squares, followed by another array of colored squares, and asked whether the two arrays were the same or different. Participants performed significantly above chance in this change-detection task (*M* = 66.6%, *t*(61) = 7.70, *p* < 0.001, *d* = .98).

The test phase consisted of 200 total trials (50 remember, 50 forget, 100 lures). Test lures were unique real-world objects that were not presented during the study phase. Subjects responded to each object by selecting one of four response buttons (Figure 1a). The experiment ended with postexperiment debriefing.

Experiment 1 can be run online here: https://maxceylab.github.io/expts/dfsolo/GeneralProcedure_mturk_1a.html.

#### Data analysis

For the old/new recognition judgment measures, we derived the signal detection measure for the area under the curve (AUC), representing memory discrimination de-confounded from potential response bias, separately for each subject and each object type. AUC takes into account reported confidence (different points along the receiver operating characteristic (ROC) curve) and provides an index of discriminability that does not depend on strong, typically untested, assumptions about the distribution of internal states—it is simply a measure of ordinal separation of the two distributions indexing the responses to targets and lures, respectively (Weidemann & Kahana, 2016). While AUC was our primary measurement, we also reported hit rate (more standard measure for directed forgetting) and *d*′ (more standard measurement for recognition memory) for comparison. Each subject's magnitude of directed forgetting was calculated as the difference of AUC for Remember items versus Forget items. We excluded subjects who performed at or below chance (50%) in terms of accuracy and/or false alarms across all Old/New reports. The full data set is available on OSF (https://osf.io/yq5nj/).

### Results

#### Directed forgetting

**Figure 2.**
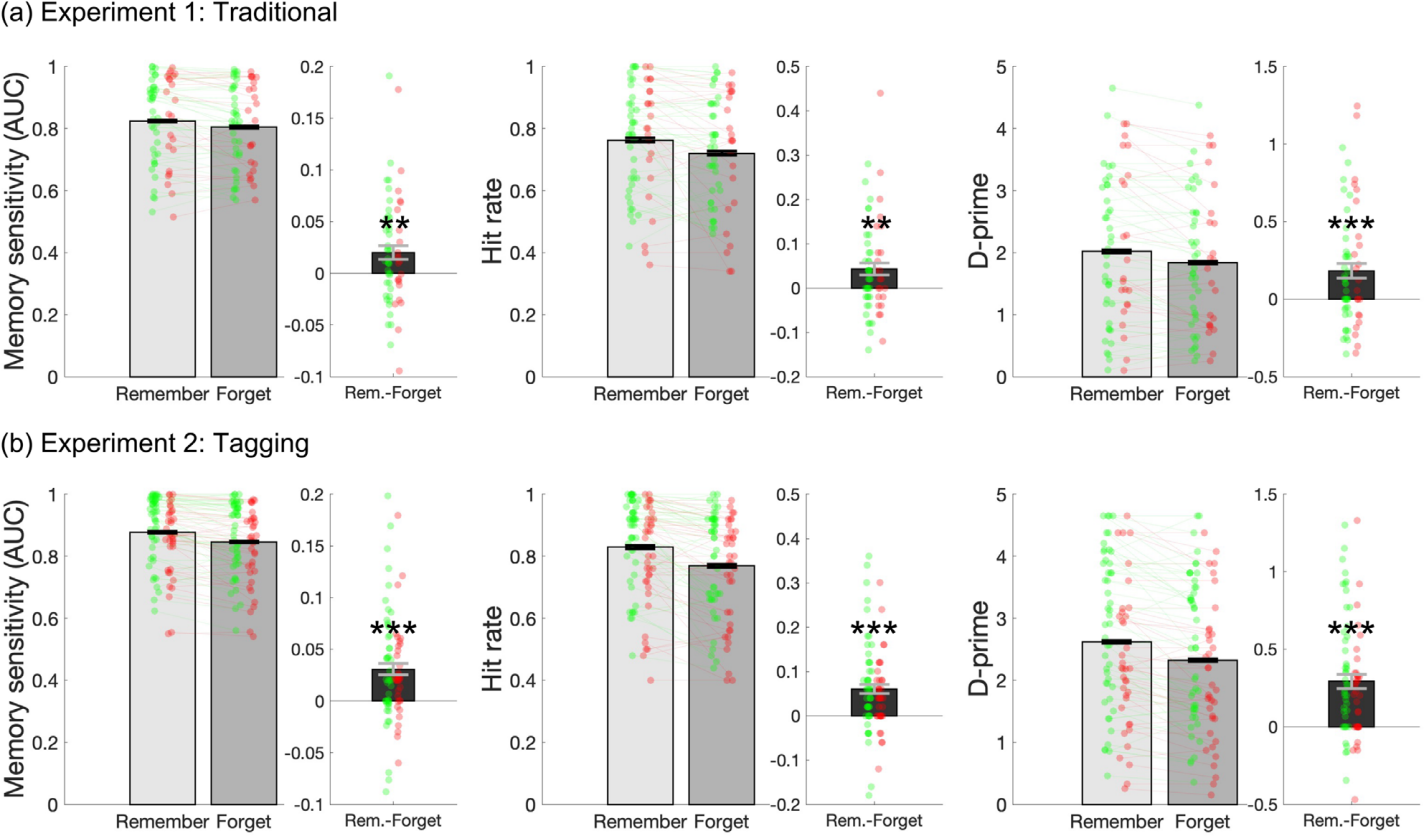
Data across AUC, hit rate, and *d*′ from (a) Experiment 1 and (b) Experiment 2. Light gray bars represent memory for pictures that were followed by a Remember cue. Dark gray bars represent memory for pictures that were followed by a Forget cue. Black bars represent the difference in memory between Remember and Forget objects (i.e., directed forgetting). Green dots represent subjects who reported believing the cue to Forget. Red dots represent subjects who reported not believing the cue to forget. Error bars represent ± 1 *SEM*. Directed forgetting was reliable across all comparisons. *p < 0.05. **p < 0.01. ***p < 0.001.

Directed forgetting was reliable in Experiment 1 (Figure 2a), with memory for remember-cued items better than memory for forget-cued items, across AUC (Remember *M* = 0.824, Forget *M* = 0.804, *t*(61) = 2.99, *p* = 0.004, *d* = .38), hit rate (Remember *M* = 0.763, Forget *M* = 0.720, *t*(61) = 2.42, *p* = 0.002, *d* = .41), and *d*′ (Remember *M* = 2.018, Forget *M* = 1.836, *t*(61) = 3.78, *p* < 0.001, *d* = .48). To reiterate, the size of directed forgetting of pictures measured in hit rate is 4.3%.

#### Postexperiment debriefing

In the postexperiment debriefing, a large portion of subjects reported not trusting the Forget instruction (37%, consistent with other studies soliciting subjects’ rates of disbelief in the instruction to forget (Foster & Sahakyan, 2011; Scotti & Maxcey, 2021). The subjects who reported distrust of the Forget instruction still showed reliable directed forgetting across hit rate (Remember *M* = .768, Forget *M* = .710, *t*(22) = 2.23, *p* = 0.028, *d* = .46) and *d*′ (Remember *M* = 2.08, Forget *M* = 1.84, *t*(22) = 2.53, *p* = 0.019, *d* = .53) but not AUC (Remember *M* = .823, Forget *M* = .804, *t*(22) = 1.59, *p* = 0.126, *d* = .33). There was no significant difference in directed forgetting between subjects who reported believing and disbelieving the cues (using independent samples *t* tests across AUC, *t*(60) = 0.12, *p* = 0.906, *d* = .03, JZS_NULL_= 3.73; hit rate, *t*(60) = 0.85, *p* = 0.397, *d* = .22, JZS_NULL_ = 2.78; or *d*′, *t*(60) = 0.91, *p* = 0.368, *d* = .24, JZS_NULL_ = 2.66). These results replicate Scotti and Maxcey (2021), but here they do so using the classic two-phase directed forgetting paradigm. To avoid overinterpreting these results, we discuss them further in the General discussion.

### Discussion

In Experiment 1, we observed robust directed forgetting effects using real-world objects and the traditional item-method directed forgetting procedure. Such results are informative because they corroborate previous observations of directed forgetting and generalize the effect to real-world objects. We discuss how different stimulus types interact with directed forgetting by comparing the relative magnitudes of directed forgetting in the General discussion.

## Experiment 2: Tagging in directed forgetting

In Experiment 2, we aim to replicate the results of Experiment 1 using a modified directed forgetting procedure where participants have to additionally specify the associated tag for Old items (Old-Remember or Old-Forget). This modification should help to ensure that participants are not confused about how to respond to Old items that they believe were supposed to be forgotten and may further reduce demand characteristics where participants could believe that the experimenter wants them to show worse memory performance for Forget items. A replication of Experiment 1 with finer control over response confusion and/or demand characteristics can help to ensure that our previous results are reproducible and generalizable.

### Methods

#### Participants

Data were collected from 168 new subjects. We aimed for more subjects in Experiment 2 to facilitate analysis of the postexperiment survey. Quality assurance trimming left 85 subjects remaining (mean age: 29.96 [*SD* 3.37], 40 female, 44 male, 1 nonbinary) who were included in analyses below.

#### Procedure

Experiment 2 was identical to Experiment 1 with the following exceptions. Two additional response buttons were added to reduce demand characteristics and response confusion (Figure 1b). The postexperiment debriefing was revised to remove a question rendered irrelevant due to the additional response buttons. Participants in Experiment 2 performed significantly above chance in the change detection filler task (*M* = 61.8%, *t*(84) = 5.04, *p* < 0.001, *d* = .55).

Experiment 2 can be run online here: https://maxceylab.github.io/expts/dfsolo/GeneralProcedure_mturk_1b.html.

#### Data analysis

We analyzed Experiment 2 in the same manner as Experiment 1. The intention with the tagging manipulation was to ensure proper understanding of task instructions and reduce demand characteristics, so we discarded the associated tag reported during analysis such that Old-Remember and Old-Forget were evaluated as the same response. Still, such tagging reports might contain important information, so we report the percentage of responses given for each response option in Table 1.

**Table 1.** Proportion of trials in which participants pressed each of the four response options in Experiment 1 (Old Sure, Old Unsure, New Sure, New Unsure) and each of the six possible response options in Experiment 2 (Old Remember Sure, Old Remember Unsure, Old Forget Sure, Old Forget Unsure, New Sure, New Unsure). Proportions for Experiments 2 and 3 were further split by whether the subjects reported trust or distrust in the cued instructions to forget, as gauged by the postexperiment survey.

	Old sure	Old unsure	New sure	New unsure		
Experiment 1	0.359	0.101	0.325	0.215		
	Old Remember Sure	Old Remember Unsure	Old Forget Sure	Old Forget Unsure	New Sure	New Unsure
Experiment 2	0.157	0.034	0.162	0.108	0.375	0.164
Experiment 2 (trusted cues, *n* = 48)	0.159	0.031	0.177	0.097	0.375	0.162
Experiment 2 (distrusted cues, *n* = 37)	0.154	0.038	0.142	0.123	0.375	0.168
Experiment 3	0.144	0.059	0.121	0.157	0.252	0.267
Experiment 3 (trusted cues, *n* = 66)	0.147	0.051	0.128	0.157	0.251	0.266
Experiment 3 (distrusted cues, *n* = 22)	0.134	0.086	0.095	0.157	0.256	0.272

### Results

#### Directed forgetting

Directed forgetting was reliable (Figure 2b), with memory for Remember cued items better than memory for Forget cued items, across AUC (Remember *M* = 0.877, Forget *M* = 0.846, *t*(84) = 5.43, *p* < 0.001, *d* = .59), hit rate (Remember *M* = 0.829, Forget *M* = 0.769, *t*(84) = 5.67, *p* < 0.001, *d* = .62), and *d*′ (Remember *M* = 2.62, Forget *M* = 2.33, *t*(84) = 6.37, *p* < 0.001, *d* = .69). To reiterate, the size of directed forgetting of pictures measured in hit rate is 6.0%.

#### Postexperiment debriefing

In the postexperiment debriefing, 44% of subjects (*n* = 37/85) reported not trusting the Forget instruction. Subjects who reported trust of the Forget instruction showed reliable directed forgetting across AUC (Remember *M* = .900, Forget *M* = .863, *t*(47) = 4.00, *p* < 0.001, *d* = .58), hit rate (Remember *M* = .858, Forget *M* = .790, *t*(47) = 4.33, *p* < 0.001, *d* = .62), and *d*′ (Remember *M* = 2.86, Forget *M* = 2.52, *t*(47)=4.87, *p* < 0.001, *d* =.70). Subjects who reported distrust of the Forget instruction also showed reliable directed forgetting across AUC (Remember *M* = .851, Forget *M* = .824, *t*(36) = 3.74, *p* < 0.001, *d* = .61), hit rate (Remember *M* = .792, Forget *M* = .742, *t*(36) = 3.74, *p* < 0.001, *d* = .61), and *d*′ (Remember *M* = 2.30, Forget *M* = 2.07, *t*(36) = 4.27, *p* < 0.001, *d* = .70). There was no significant difference in directed forgetting between subjects who reported believing and disbelieving the cues (using independent samples *t* tests across AUC, *t*(83) = 0.52, *p* = 0.602, *d* = .11, JZS_NULL_ = 3.90; hit rate, *t*(83) = 0.87, *p* = 0.388, *d* = .19, JZS_NULL_ = 3.15; or *d*′, *t*(83) = 1.14, *p* = 0.258, *d* = .25, JZS_NULL_ = 2.49). As with Experiment 1, to avoid overinterpreting these results, we discuss them further in the General discussion.

The difference in overall accuracy (disregarding tag specificity) between subjects who believed (*M* = 84.8%) versus disbelieved (*M* = 79.5%) the forget cues was not significant (*t*(83) = 1.95, *p* = 0.055, *d* = .43, JZS_ALT_ = 1.18). Regarding tag-specific reports, there was a significant difference in the proportion of correct Old-Remember reports between subjects who believed (*M* = 82.0%) versus disbelieved (*M* = 69.5%) the cues (*t*(83) = 3.09, *p* = 0.003, *d* = .68). There was no significant difference in the proportion of correct Old-Forget reports between subjects who believed (*M* = 64.6%) versus disbelieved (*M* = 56.4%) the cues (*t*(83) = 1.81, *p* = 0.073, *d* = .40, JZS_NULL_ = 1.06). These results seem to suggest that subjects who reported disbelief in the cues to Forget suffered from overall poorer memory performance for Old-Remember items but in way that did not interact with directed forgetting effects.

### Discussion

In Experiment 2, we again observed robust directed forgetting effects using real-world objects, this time using an item-method directed forgetting procedure where participants needed to specify whether Old items were presented alongside the Remember or Forget instruction. These results validate the results of Experiment 1 and confirm that directed forgetting can operate across everyday objects without response confusion or demand characteristics as probable alternative explanations. While this experiment attenuates demand characteristics, it does not eliminate them. Replicating these results in a laboratory setting with converging evidence from response-free neurobiological measures might be one way to more thoroughly investigate directed forgetting in the absence of demand characteristics.

## Experiment 3: Pictures versus words in the laboratory

The use of a standard item-method procedure, as opposed to modified or untraditional procedures, allows for comparison of effect sizes across item-method directed forgetting experiments. Here we observed a directed forgetting effect of 6% (hit rate) using the traditional directed forgetting procedure with pictures of everyday objects. This is a relatively weak effect compared to directed forgetting effects of approximately 20% to 30% using words (Basden et al., 1993; MacLeod, 1999; Quinlan et al., 2010), 9% to 12% using abstract symbols (Hourihan et al., 2009), 8% using line drawings (Quinlan et al., 2010), and 6% using real-world scenes (Hauswald & Kissler, 2008). The large differences in directed forgetting magnitudes across studies suggests that long-term memory susceptibility to directed forgetting depends on the stimulus type, with verbal stimuli being most susceptible, artificial visual stimuli being moderately susceptible, and naturalistic visual stimuli (like everyday objects and scenes) being least susceptible.

One may argue that the reason for the reduced effect size for directed forgetting of everyday objects in the present study (relative to previous studies using words) is due to other factors that vary between studies. One likely alternative explanation is that the present work was run online, potentially increasing error variance, whereas the studies to which we referred above were run in controlled laboratory conditions. To test the alternative explanation, that the large-magnitude difference between pictures and words is due to the present study with pictures being conducted online and the previous studies using words were conducted in person, we replicated Experiment 2 in a controlled laboratory setting using a within-subjects design to test directed forgetting of both pictures and words.

### Methods

#### Participants

Data were collected from 104 new subjects. We aimed for an equivalent number of subjects in Experiment 3 as in Experiment 2 (*n* = 85). Quality assurance trimming left 86 subjects remaining (mean age: 19.22 [*SD* 1.07], 63 female, 22 male, 1 preferred not to answer) who were included in analyses below.

#### Procedure

Experiment 3 was identical to Experiment 2 with the following exceptions. The study phase included 200 total trials (100 pictures, 100 words; each stimulus type included 50 stimuli that were cued to remember and 50 cued to forget). Each object was displayed for 2 seconds, followed by the word *remember* or *forget* displayed for 2.5 seconds, directing them to either forget an object or remember the object for a later memory test. The words clearly differed from the remember or forget cues because the words were presented in all caps and a larger font size. The test phase consisted of 400 total trials (200 of each stimulus type, further subdivided by 50 remember, 50 forget, 100 lures). Pictures and words were randomly intermixed throughout the study and test phases. Experiment 3 can be run online here: https://maxceylab.github.io/expts/dfsolo/GeneralProcedure_3.html.

### Results

#### Directed forgetting of pictures

**Figure 3.**
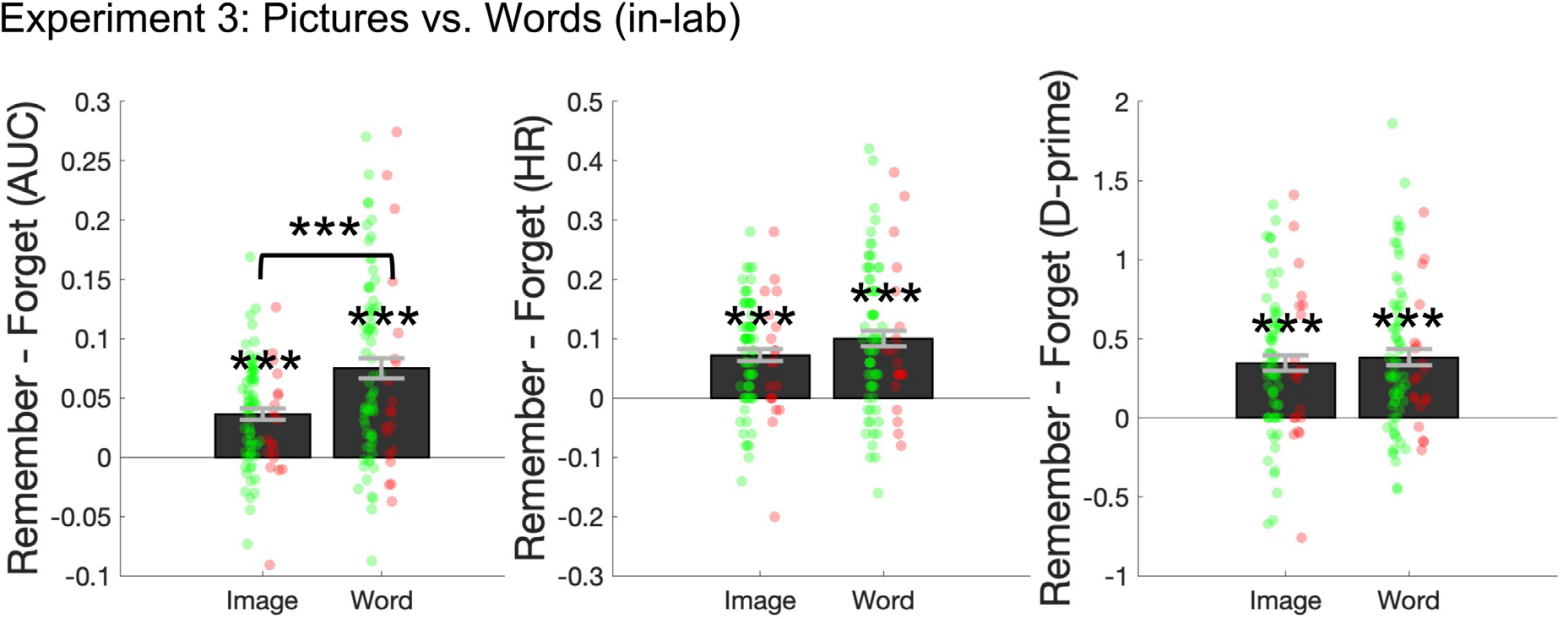
Directed forgetting (memory for Remember – memory for Forget) across AUC, hit rate, and *d*′ from Experiment 3. Green dots represent subjects who reported believing the cue to Forget. Red dots represent subjects who reported not believing the cue to forget. Error bars represent ± 1 *SEM*. Directed forgetting was reliable across all comparisons, ***p < 0.001. The difference in directed forgetting between picture and words was only reliable when using AUC.

Directed forgetting of pictures was reliable (Figure 3), with memory for Remember cued items better than memory for Forget cued items, across AUC (Remember *M* = 0.896, Forget *M* = 0.859, *t*(85) = 7.52, *p* < 0.001, *d* = .81), hit rate (Remember *M* = 0.834, Forget *M* = 0.762, *t*(85) = 7.14, *p* < 0.001, *d* = .94), and *d*′ (Remember *M* = 2.63, Forget *M* = 2.28, *t*(85) = 7.03, *p* < 0.001, *d* = .76).

#### Directed forgetting of words

Directed forgetting of words was reliable, with memory for Remember cued items better than memory for Forget cued items, across AUC (Remember *M* = 0.764, Forget *M* = 0.689, *t*(85) = 8.76, *p* < 0.001, *d* = .94), hit rate (Remember *M* = 0.735, Forget *M* = 0.634, *t*(85) = 7.55, *p* < 0.001, *d* = .81), and *d*′ (Remember *M* = 1.32, Forget *M* = 0.935, *t*(85) = 7.60, *p* < 0.001, *d* = .82).

#### Interaction between directed forgetting of pictures and words

Directed forgetting of pictures was reliably larger than words when measured with AUC (average magnitude of forgetting difference between pictures × words: 0.039, *t*(85) = 4.77, *p* < 0.001, *d* = .51), marginally larger when measured with hit rate (average magnitude of forgetting difference between pictures × words: 0.028, *t*(85) = 1.98, *p* = 0.0505, *d* = .21), and not reliably different when measured using *d*′ (average magnitude of forgetting difference between pictures × words: 0.039, *t*(85) = 0.68, *p* = 0.500, *d* = .07).

### Discussion

In Experiment 3, we again observed robust directed forgetting effects using real-world objects, as well as directed forgetting of words, this time in a controlled laboratory setting. The directed forgetting effect was larger for pictures than words when using our primary measure of AUC, marginally larger when measuring hit rate, and not significant when measuring *d*′. Average directed forgetting hit rate for pictures was 7.2% (comparable to 6% in Experiment 2) and average directed forgetting hit rate for words was 10.0% (smaller than expected 20–30% found in other studies; Basden et al., 1993; MacLeod, 1999; Quinlan et al., 2010).

## General discussion

In the present study, we asked whether directed forgetting from visual long-term memory can be predicted in an item-method directed forgetting procedure using pictures of everyday objects (Corenblum et al., 2020; Quinlan et al., 2010; Thompson et al., 2011). Directed forgetting is typically studied using verbal items like everyday words, with far less research devoted to understanding how this effect generalizes to other stimulus types such as visual images. Critically, to our knowledge, directed forgetting (using the standard item-method procedure) has never been previously observed with everyday visual objects. Here we measured directed forgetting across three experiments that instructed subjects to remember or forget each object. The first experiment used a traditional item-method directed forgetting procedure, the second experiment added “tagging” to reduce response confusion and ensure a purer measure of directed forgetting, and the third experiment incorporated word stimuli to conduct a within-subjects comparison across memoranda in a controlled laboratory setting. The difference in memory for remember- and forget-cued items was reliable in all experiments, demonstrating that a typical subject population is able to exert voluntary control over pictures of everyday objects stored in visual memory.

### Postexperiment debriefing

We implemented a postexperiment survey following the traditional and tagging conditions probing subjects’ strategy in these conditions. We found statistically indistinguishable directed forgetting across subjects who did and did not believe the forget-cued items could truly be forgotten.

Before discussing debriefing results, we address potential limitations of this method.^2^ First, by querying subjects’ belief in the instruction to forget following the final memory test, they may be influenced by demand characteristics or hindsight bias to report disbelief in the cues. Second, skepticism of the forget cue does not necessarily translate into subjects reinterpreting the forget cue as a remember cue. Indeed, as pointed out by MacLeod (2012), “Directed forgetting may be a misnomer. For the item method, it would appear that people simply do not learn the F[orget] items as well as the R[emember] items: Obeying instructions, they give F[orget] items less attention and less rehearsal, resulting in weaker learning.”

Although we agree with the aforementioned skepticism of the postexperiment survey, we struggle with the logic that subjects in a directed forgetting paradigm can be trusted to accurately follow the cues to forget but not trusted to accurately respond to postexperiment debriefing. Therefore, while interpreting the survey results with caution, we interpret statistically indistinguishable performance between subjects who did and did not believe the cues to indicate one of three outcomes. First, it may be that the limitations mentioned above are correct and that querying subjects in postexperiment debriefing is uninterpretable. This outcome seems unlikely based on the fact that subjects who trusted the cues to forget showed improved accuracy on Old-Remember reports, perhaps consistent with the idea that these subjects faced reduced overall memory load. Second, directed forgetting effects may reflect solely upregulation of remember-cued items (Tozios & Fukuda, 2020). Third, it may be that directed forgetting involves a Stroop-like automaticity, where because the cues are words and reading is automatic (Stroop, 1935), subjects cannot help but implement the cues to some degree. Ongoing work in our laboratory is testing this automaticity hypothesis of directed forgetting.

### Directed forgetting in working memory versus long-term memory

Here we limit our conclusions to attentional control over the contents of long-term memory. However, it is possible that there are differences between directed forgetting of working memory versus long-term memory contents or that processes involved in directed forgetting in working memory contribute to long-term directed forgetting (Dames & Oberauer, 2022). Ongoing work by Hannah Dames and colleagues is testing whether the mechanisms driving directed forgetting of information held in working memory and long-term memory are dissociable (Dames & Oberauer, 2022; Popov & Dames, 2022).

### Theoretical implications

Here we consider how the current results can be accommodated by selective rehearsal mechanisms, which is the prevailing theoretical account of directed forgetting where directed forgetting effects are thought to occur because cognitive control is exerted to prevent rehearsal of items that are cued to forget (Anderson & Hanslmayr, 2014; Chiu et al., 2021) while items that are cued to remember are selectively rehearsed to facilitate their transfer into long-term memory (Bjork, 1972; Johnson, 1994; Sheard & MacLeod, 2005). The selective rehearsal account leaves open the possibility that the actual strength of the forget-cued item is not lowered (MacLeod, 2012; Tozios & Fukuda, 2020). For example, Tozios and Fukuda (2020) demonstrated that directed forgetting effects may be evidence of solely upregulation of remember-cued items rather than downregulation of forget-cued items. Future work should address whether forget-cued pictures show evidence of true forgetting, perhaps using electrophysiological indices of visual long-term memory (Megla et al., 2021) along the lines of electrophysiological studies of directed forgetting with words (Burgess et al., 2017; Taylor & Hamm, 2018).

### Conclusions

In the present study, we implemented three different directed forgetting procedures using pictures of everyday objects, varying the instructions to the subjects and the available responses. Across all experiments, we reliably observed volitional control over pictures of everyday objects stored in visual long-term memory. These results validate the standard item-method directed forgetting procedure as a useful task to observe voluntary control of memory using ecologically valid visual stimuli. This evidence for directed forgetting effects with everyday objects using a standardized procedure previously shown to generalize across verbal memoranda and a few distinct types of visual memoranda (i.e., abstract symbols, line drawings, scenes) contributes to this growing memory literature by showing how directed forgetting is a robust, generalizable phenomenon and how the use of a standardized procedure allows for easy comparison of directed forgetting effects across modalities and clinical populations.

We remain agnostic as to whether the volitional control seen here is driven by upregulation (Tozios & Fukuda, 2020) or downregulation (Anderson, 2005; Anderson & Hanslmayr, 2014; Basden, 1996; Brandt et al., 2013; Paz-Caballero et al., 2004; Rae et al., 2015; Rizio & Dennis, 2013; Wylie et al., 2008; Yang et al., 2016). Until the direction of this effect is resolved, the potential misnomer of directed forgetting to suggest volitional downregulation of long-term memory may lead to inappropriate application of basic research to clinical applications (e.g., posttraumatic stress disorder, obsessive-compulsive disorder) and easily avoidable confusion regarding the importance of directed forgetting to understanding inhibitory control. This should be considered in conjunction with the less controversial conclusion that attentional control is involved with volitional forgetting, likely explained by selective rehearsal (i.e., upregulation) of remember cued items (Anderson & Hanslmayr, 2014; Chiu et al., 2021). We suggest that clinically oriented studies may confidently use directed forgetting tasks to study long-term memory in relation to general attentional control capacities, but not inhibitory/suppression deficits, between control and clinical populations.

## Footnotes

1 Effect sizes in this meta-analysis were calculated as standardized mean change scores. See Stramaccia et al. (2021) for more details.

2 We thank Tracy Taylor for raising these points in a gracious, encouraging, and thoughtful previous review.
